# Supplementary material for: Phenotype switching in a global method for agent-based models of biological tissue
Source: PLoS One. 2023 Feb 13;18(2):e0281672. doi: 10.1371/journal.pone.0281672 (PMC9925070; doi:10.1371/journal.pone.0281672)
Supplement: S1 Algorithm — S: set of all lattice sites. A: set of all agents interacting with the molecule. (PDF) [file pone.0281672.s001.pdf]

Algorithm S1 Molecular dynamics in the local method. $\mathcal{S}$: set of all lattice sites. $\mathcal{A}$: set of all agents interacting with the molecule.

for $s \in \mathcal{S}$ **do**
  if s is perivascular **then**
    Update concentration at s following PK
  end if
end for
Solve diffusion PDE on the domain
for $a \in \mathcal{A}$ **do**
  Molecular exchange between a and the free concentration at the corresponding lattice site
end for
for $a \in \mathcal{A}$ **do**
  Intracellular signaling within a
end for
