# Supplementary material for: Phenotype switching in a global method for agent-based models of biological tissue
Source: PLoS One. 2023 Feb 13;18(2):e0281672. doi: 10.1371/journal.pone.0281672 (PMC9925070; doi:10.1371/journal.pone.0281672)
Supplement: S2 Algorithm — R: set of all regions. (PDF) [file pone.0281672.s002.pdf]

**Algorithm S2** Molecular dynamics in the global method. $\mathcal{R}$: set of all regions.

**for** $r \in \mathcal{R}$ **do**
    **if** $r$ is perivascular **then**
        Update concentration at $r$ following PK
    **end if**
**end for**
Solve diffusion ODE between regions
Compute average internal molecular concentrations within each region
**for** $r \in \mathcal{R}$ **do**
    Molecular exchange between agents in $r$ and the free concentration in $r$ using average concentrations in both
**end for**
**for** $r \in \mathcal{R}$ **do**
    Intracellular signaling using the average intracellular concentrations, applying the result to all agents in $r$
**end for**
