# Supplementary material for: Phenotype switching in a global method for agent-based models of biological tissue
Source: PLoS One. 2023 Feb 13;18(2):e0281672. doi: 10.1371/journal.pone.0281672 (PMC9925070; doi:10.1371/journal.pone.0281672)
Supplement: S1 Table — (PDF) [file pone.0281672.s003.pdf]

Variable	Description
$C = C(t, \mathbf{x})$	Concentration of substrate at time t and location $\mathbf{x}$ in the microenvironment
$I = I(t)$	Internalized concentration of substrate in a single agent at time t

Table S1. State variables in the local method.
