# Supplementary material for: Phenotype switching in a global method for agent-based models of biological tissue
Source: PLoS One. 2023 Feb 13;18(2):e0281672. doi: 10.1371/journal.pone.0281672 (PMC9925070; doi:10.1371/journal.pone.0281672)
Supplement: S2 Table — (PDF) [file pone.0281672.s004.pdf]

Variable	Description
$C = C_i(t)$	Concentration of substrate at time t in region i in the microenvironment
$I = I_{ik}(t)$	Average internalized concentration of substrate within all agents of type k in region i at time t

Table S2. State variables in the global method.
