# Supplementary figures and images for: Phenotype switching in a global method for agent-based models of biological tissue
Source: PLoS One. 2023 Feb 13;18(2):e0281672. doi: 10.1371/journal.pone.0281672 (PMC9925070; doi:10.1371/journal.pone.0281672)

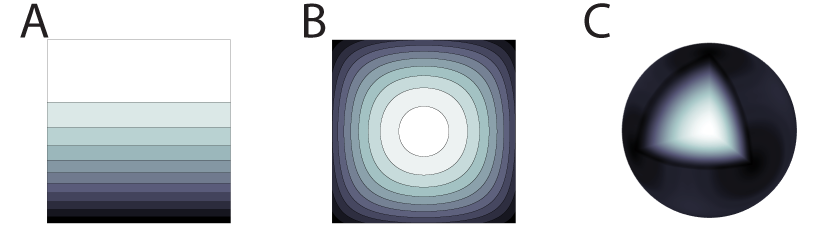

Supplement: S1 Fig — All panels show a snapshot from a simulation without any cells after a steady state has been achieved. A. Blood vessels are located on the bottom of the 2D microenvironment. B. Blood vessels are located along the entire boundary of the 2D microenvironment. C. Blood vessels are located in a spherical shell centered in the microenvironment. Concentrations are shown within this shell. The first octant relative to the center of the microenvironment has been cut away to show the concentration in the interior. (TIF) [file pone.0281672.s005.tif]

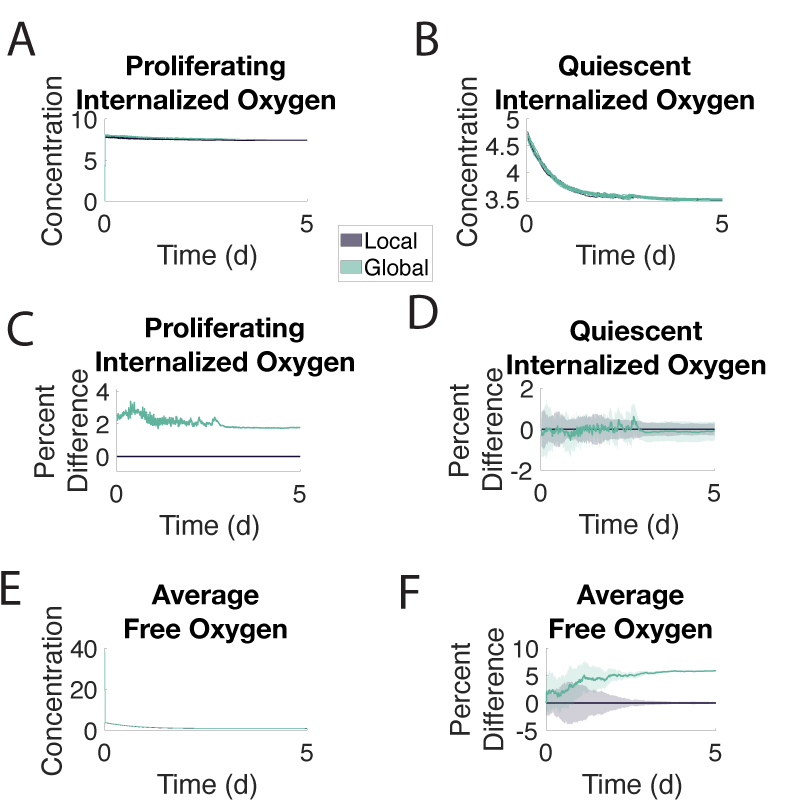

Supplement: S2 Fig — Shaded areas represents ±1 SD from the mean. A. Average internalized oxygen concentration in proliferating cells. B. Average internalized oxygen concentration in quiescent cells. C-D. Percent difference between the two methods in A-B above, respectively. E. Average oxygen concentration throughout the microenvironment. F. Percent difference between the two methods in E. (TIF) [file pone.0281672.s006.tif]

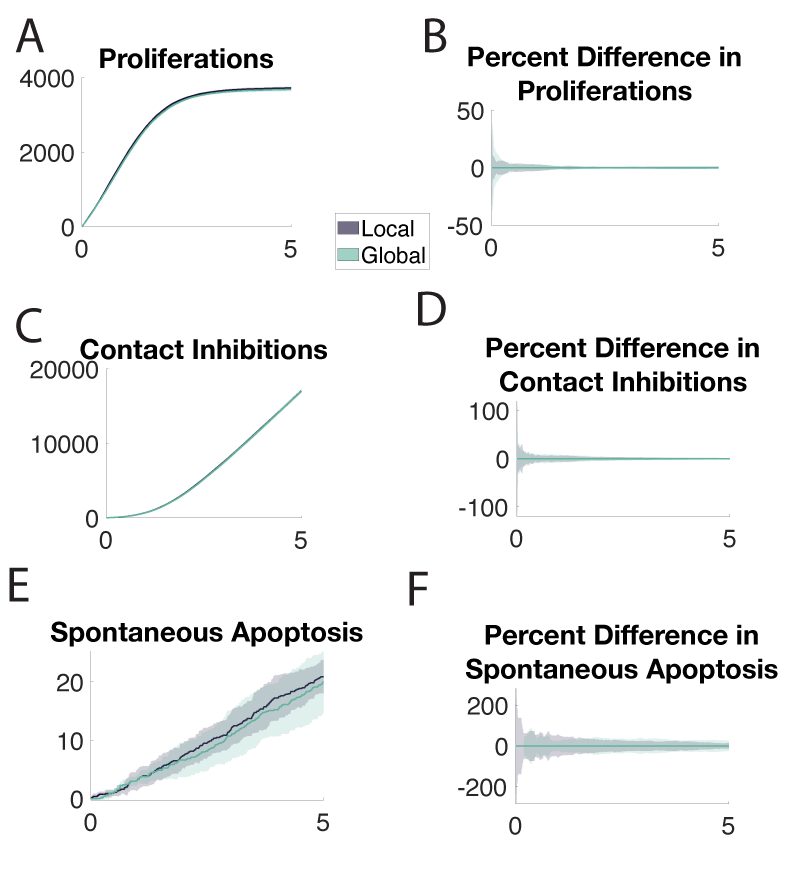

Supplement: S3 Fig — A. Number of proliferations. B. Percent difference between the two methods in A. C. Number of contact inhibitions. D. Percent difference between the two methods in C. E. Number of apoptotic events. (TIF) [file pone.0281672.s007.tif]

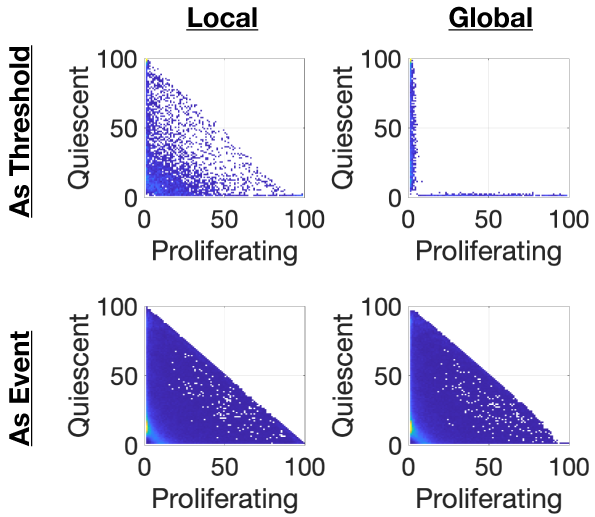

Supplement: S4 Fig — Each panel shows a heatmap of the composition of all regions throughout the simulation and across all samples. The x-axis indicates how many proliferating cells are in the region. The y-axis indicates how many quiescent cells are in the region. The heatmap is restricted to only regions that contained at least one of both cell types, otherwise the values along the axes would dominate the values shown here. Top row: quiescence is determined based on a threshold value for the internalized oxygen. Bottom row: quiescence is an event that cells can stochastically undergo based on internalized oxygen. (TIF) [file pone.0281672.s008.tif]

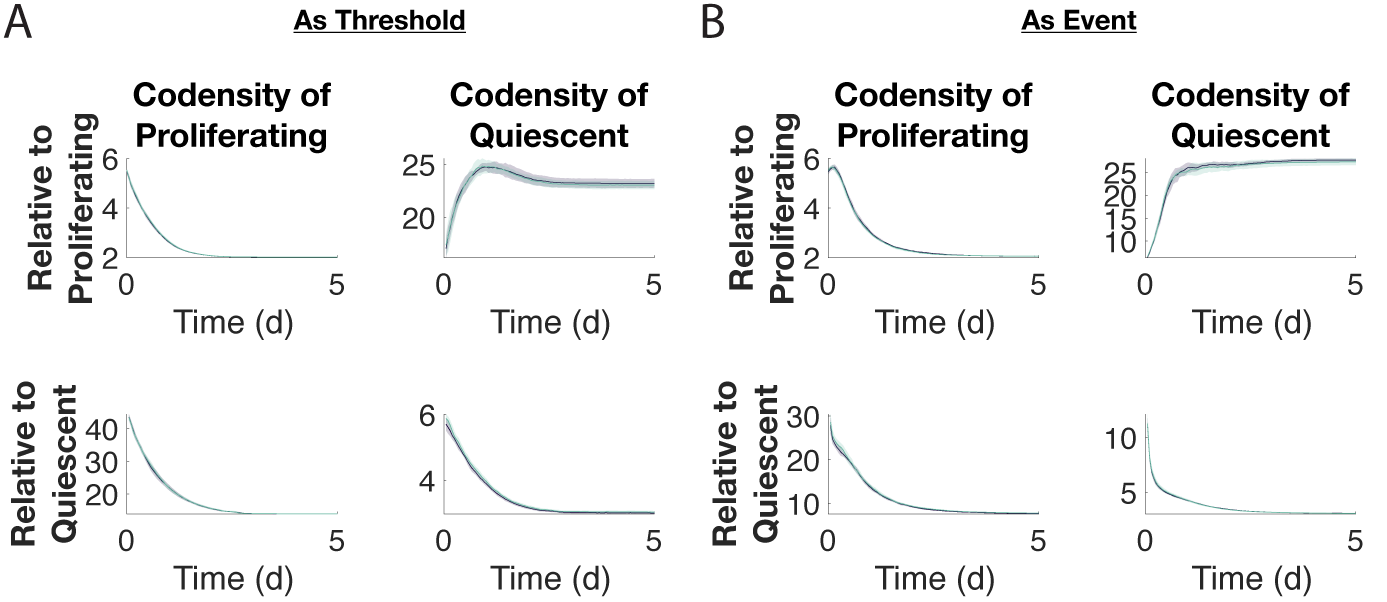

Supplement: S5 Fig — Each set of axes shows the codensity of one cell type (in the column) relative to another (in the row) for both the local and global methods. Shaded area represents ±1 SD from the mean. A. Quiescence modeled as a threshold. B. Quiescence modeled as an event. (TIF) [file pone.0281672.s009.tif]

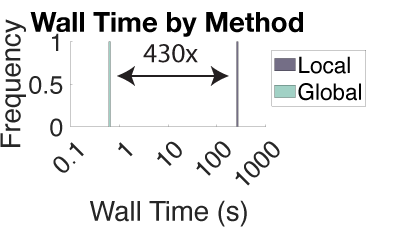

Supplement: S6 Fig — Simulations were ran until t = 2 min. One sample was run for each method. (TIF) [file pone.0281672.s010.tif]

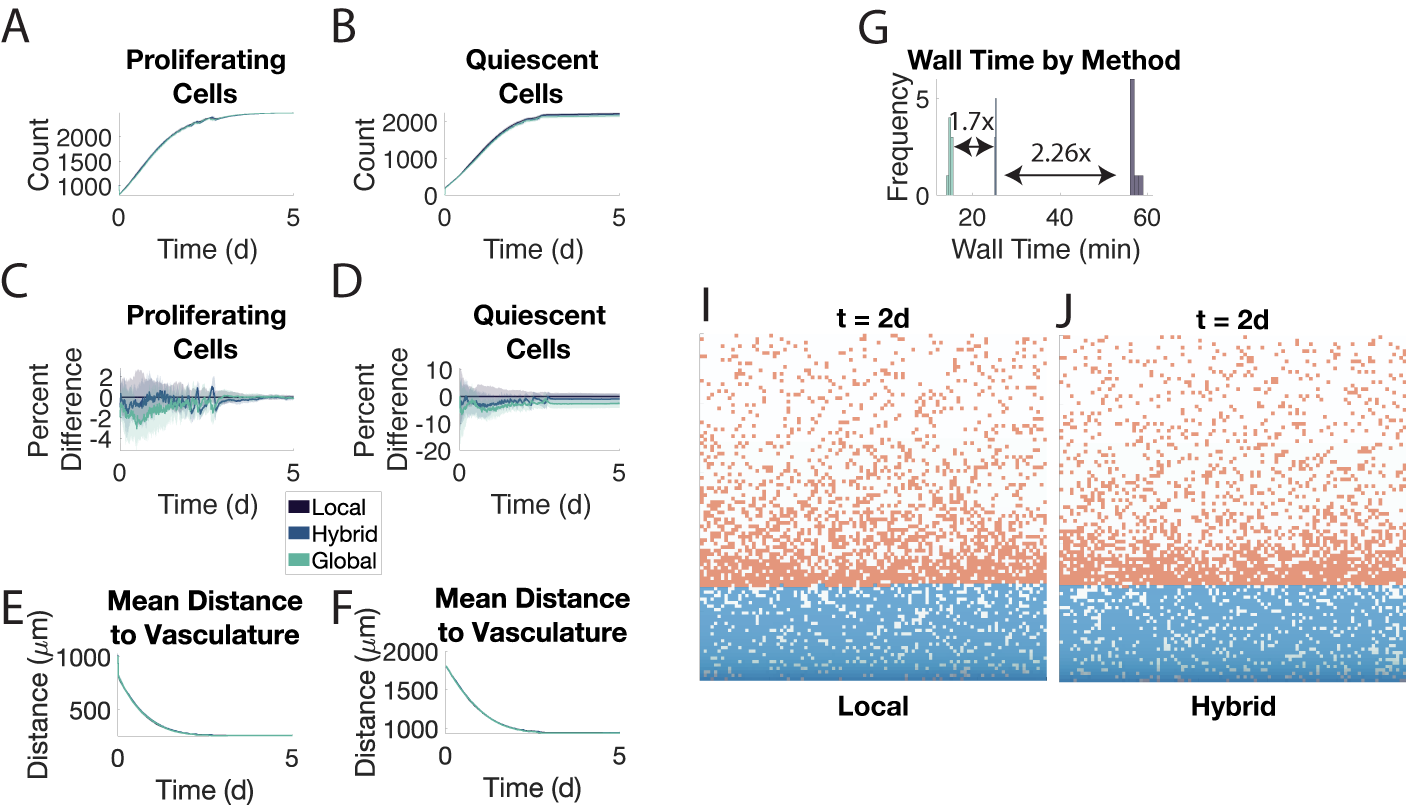

Supplement: S7 Fig — Compare to Fig 2. (TIF) [file pone.0281672.s011.tif]

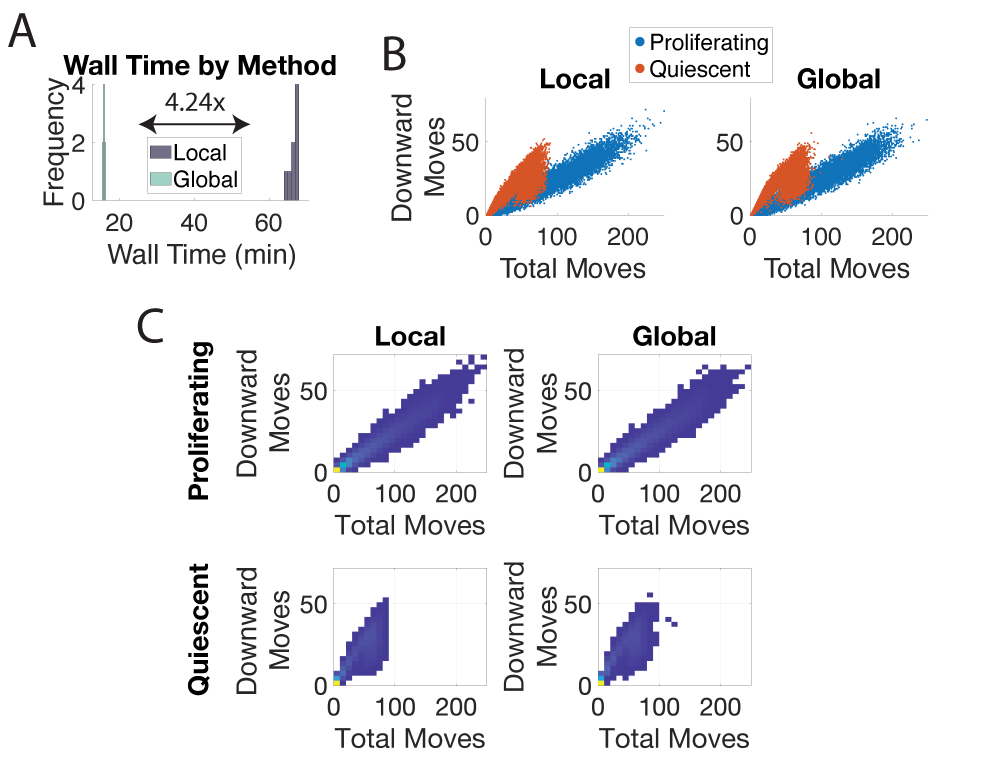

Supplement: S8 Fig — A. Wall time distributions for the two methods. B. Comparison of movements downward (along oxygen gradient) against all movement for both cell types across both methods. Each point represents all movements within one continuous time window in which a agent is in a constant state. C. Heat maps of the four scatter plots shown in B. (TIF) [file pone.0281672.s012.tif]
